# A Circularly Polarized Implantable Rectenna for Microwave Wireless Power Transfer

**DOI:** 10.3390/mi13010121

**Published:** 2022-01-12

**Authors:** Chao Xu, Yi Fan, Xiongying Liu

**Affiliations:** 1 School of Electronic and Information Engineering, South China University of Technology, Guangzhou 510640, China; students_china@163.com; 2 National Engineering Technology Research Center for Mobile Ultrasonic Detection, Guangzhou 510640, China; 3 School of Electronic and Information, Guangdong Polytechnic Normal University, Guangzhou 510665, China; hnanfy@163.com

**Keywords:** circular polarization, implantable antenna, rectenna, rectifier, RF energy harvesting, wide band, wireless power transfer

## Abstract

A circularly polarized implantable antenna integrated with a voltage-doubled rectifier (abbr., rectenna) is investigated for microwave wireless power transfer in the industrial, scientific, and medical (ISM) band of 2.4–2.48 GHz. The proposed antenna is miniaturized with the dimensions of 7.5 mm × 7.5 mm × 1.27 mm by etching four C-shaped open slots on the patch. A rectangular slot truncated diagonally is cut to improve the circular polarization performance of the antenna. The simulated impedance bandwidth in a three-layer phantom is 30.4% (1.9–2.58 GHz) with |S_11_| below −10 dB, and the 3-dB axial-ratio bandwidth is 16.9% (2.17–2.57 GHz). Furthermore, a voltage-doubled rectifier circuit that converts RF power to DC power is designed on the back of the antenna. The simulated RF-to-DC conversion efficiency can be up to 45% at the input power of 0 dBm. The proposed rectenna was fabricated and measured in fresh pork to verify the simulated results and evaluate the performance of wireless power transfer.

## 1. Introduction

Implantable medical devices (IMDs), which send information about human physiology to external medical receivers wirelessly through the implanted antenna and allow doctors to diagnose and treat patients conveniently [1], are now widely used in various medical scenarios, such as pacemakers [2], nerve stimulators [3], and biosensors [4]. To supply power to IMDs properly inside the human body, lithium batteries are always equipped [5,6]. Nevertheless, expensive surgical operations are needed to change the life-limited batteries, and also cause the patient to suffer pain. Due to wireless power transfer (WPT), it has the potential to extend the lifetime of IMDs and alleviate the operation pains of patients. Depending on the different application scenarios [7], a few WPT works including near-field coupling [8,9,10] and microwave radiation [11,12,13,14,15] have been developed. Compared with microwave radiation, the near-field coupling system is usually shorter in transmission distance and larger in size. Hence, WPT based on microwave radiation may be a better approach for the tiny IMDs. 

In the receiving element of the microwave WPT system, an implantable antenna for receiving RF power and a rectifier circuit for converting RF power into DC power are needed. Implantable linearly polarized rectennas for far-field microwave WPT are designed and analyzed in [11,12,13]. A triple-band implantable rectenna with a stacked and spiral structure is proposed in [11]. Those three frequency bands could be used for data telemetry, wake-up/sleep controller, and WPT, respectively. Compact-size implantable rectennas with planar inverted-F antenna (PIFA) and a rectifier are designed in [12] and [13]. Through adding a parasitic patch [12] or commercially available gel [13] on the surface of the human body, the received power levels are enhanced by 12.9 dB and 6.2 dB, respectively. In addition to far-field microwave WPT, rectennas in [14,15] are also proposed for near-field microwave WPT to obtain high transmission efficiency.

Compared with linear polarization, circular polarization (CP) can make the position of the antenna more arbitrary and weaken the multipath effect [16,17,18,19]. In [16], a miniaturized CP implantable antenna is proposed by utilizing the capacitive loading on the radiator. To realize miniaturization by slow-wave effect, a CP implantable antenna with four LC loadings is designed in [17]. In [18], a wide-beamwidth circularly polarized implantable antenna is introduced by loading a complementary split-ring resonator (CSRR) and four C-shaped slots on the radiation patch. To extend the axial ratio (AR) bandwidth of the antenna, a wideband CP implantable antenna by exciting multiple degenerate modes is designed in [19]. For microwave WPT, a wireless power link including a CP implantable antenna is studied in [20]. The results show that the system can obtain higher DC power compared with the previous work, whereas the antenna has a narrow bandwidth.

In the wireless power transmission system, the wider the bandwidth of the antenna is, the more RF power at different frequencies the system can collect. Furthermore, broadband can also make implantable antennas more robust in the human body. In this work, a wideband CP implantable rectenna is proposed for microwave WPT operating in the ISM band of 2.4–2.48 GHz. The proposed rectenna consists of a compact CP implantable antenna and a rectifier circuit designed under the ground. By etching four C-shaped open slots, the proposed antenna is miniaturized and has a wide bandwidth. The CP purity of the proposed antenna can be optimized through a rectangular slot truncated diagonally. To establish a wireless power link and analyze the performance of power transmission, the proposed CP implantable rectenna was fabricated and tested.

## 2. Antenna Design and Discussion 

### 2.1. Antenna Design

**Figure 1:**
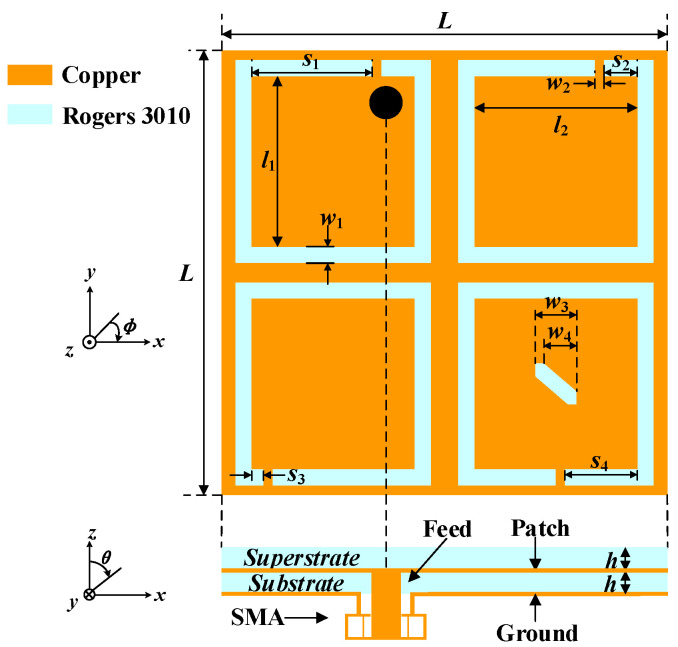
Geometry of the proposed implantable antenna.

The geometry of the proposed antenna is illustrated in Figure 1. The optimized dimensions with the electromagnetic numerical simulation software ANSYS HFSS *v*.18 are listed in Table 1. Four C-shaped open slots are etched in the four corners of the radiation patch, which can realize the miniaturization of the proposed antenna and excite a pair of degenerate modes with equal amplitude and orthogonal polarization. Furthermore, by loading a rectangular slot truncated diagonally in one of the C-shaped open slots, the CP purity of the antenna can be improved. As for the substrate and superstrate, the Rogers RO3010 (*ε*_r_ = 10.2, tan *δ* = 0.0035) with a high dielectric constant is employed to achieve miniaturization. The substrate and superstrate have the same thickness of 0.635 mm.

**Figure 2:**
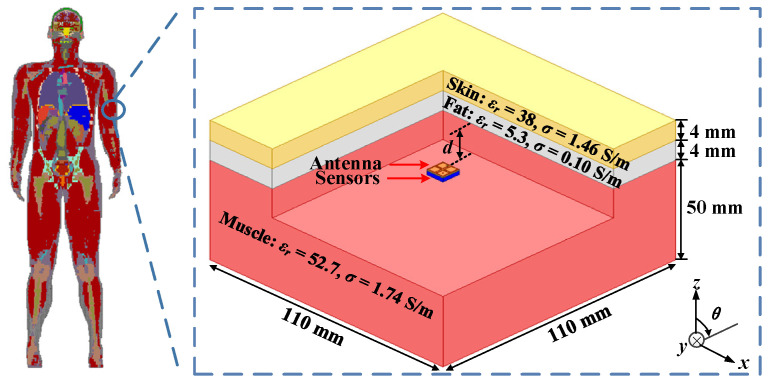
Simulation environment of the three-layer phantom model.

As shown in Figure 2, the three-layer phantom, consisting of skin, fat, and muscle, is introduced to simulate the working environment of the proposed antenna in the human body. 

**Figure 3:**
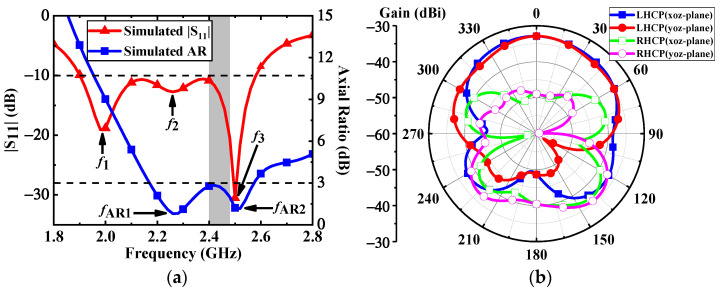
Simulated results of the proposed antenna: (**a**)|S_11_| and axial ratio, (**b**) LHCP and RHCP radiation patterns at 2.4 GHz.

Due to the fact that the electromagnetic properties of human tissues change with frequency, the relative permittivity and conductivity of the three-layer human model are set at the central frequency of 2.45 GHz [21]. The proposed antenna is implanted at a depth *d* = 4 mm in the muscle. The total depth of implantation is 12 mm. With reference to Figure 3a, the simulated impedance bandwidth is covered from 1.9 GHz to 2.58 GHz with |S_11_| below −10 dB, and the 3-dB AR bandwidth is from 2.17 GHz to 2.57 GHz. Both the impedance bandwidth and AR bandwidth coincide with each other in the ISM band of 2.4–2.48 GHz. As depicted in Figure 3b, the proposed antenna has a peak gain of –32.8 dBi at 2.4 GHz. In the *xz*- and *yz*- planes, the left-handed circular polarization (LHCP) patterns of the proposed antenna occupy the main radiation component in the main radiation direction, and its cross-polarization discrimination is about 16 dB.

### 2.2. Operating Mechanism

**Figure 4:**
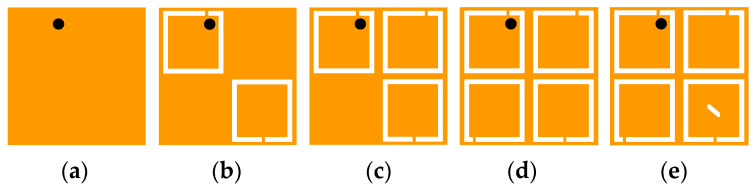
Evolution of the proposed antenna topology: (**a**) Case 1, (**b**) Case 2, (**c**) Case 3, (**d**) Case 4, and (**e**) Case 5.

**Figure 5:**
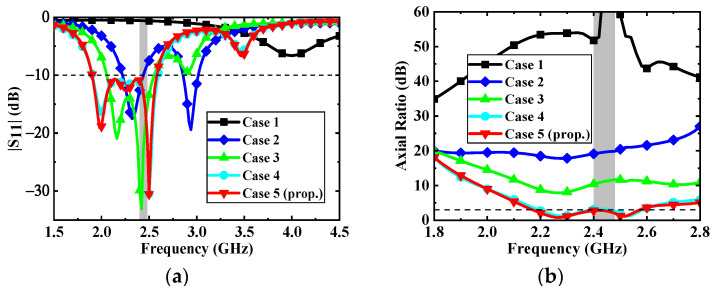
Simulated results of a different case: (**a**) |S_11_| and (**b**) Axial ratio.

To better understand the operating principle for realizing miniaturization and circular polarization, the antenna topologies are evolving from Case 1 to Case 5, as illustrated in Figure 4. The corresponding simulated results in different cases are shown in Figure 5. Initially, the antenna is based on a microstrip patch antenna. It can be seen from Case 1 that the antenna excites the fundamental resonant TM_01_ mode and resonates at 4 GHz. To lower the resonant frequency of the antenna, two C-shaped open slots are introduced along the diagonal of the patch in Case 2, extending the effective paths of the current. Hence, the resonant frequency of TM_01_ mode is shifted to 2.3 GHz, exciting another resonance at 2.9 GHz. In addition, a C-shaped open slot is further introduced in the upper right corner in Case 3 to split orthogonal degenerate modes TM_01_ (2.16 GHz) and TM_10_ (2.42 GHz). In Case 4, the impedance of the resonance at the upper frequency is further matched and the resonant frequency is reduced by adding another C-shaped open slot in the lower-left corner, thus expanding the impedance bandwidth of the antenna.

**Figure 6:**
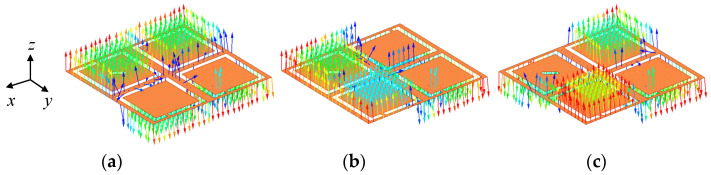
Electric field distributions of the proposed antenna: (**a**) *f*_1_ (2 GHz), (**b**) *f*_2_ (2.26 GHz) and (**c**) *f*_3_ (2.5 GHz).

**Figure 7:**
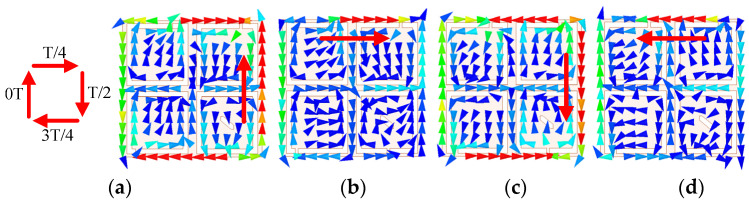
Current distributions of the proposed antenna: (**a**) *t* = 0T, (**b**) *t* = T/4, (**c**) *t* = T/2 and (**d**) *t* = 3T/4.

As can be seen from the electric field distributions in Figure 6, the resonant frequencies of *f*_1_ and *f*_2_ are orthogonal degenerate modes TM_01_ and TM_10_, respectively, and the higher resonant frequency of *f*_3_ is TM_11_ mode. By loading a rectangular slot truncated diagonally in the lower right C-shaped open slot of Case 5, the CP purity of the antenna is improved and the phase difference of TM_01_ and TM_10_ is approaching 90° for CP realization, which generates an AR minimum of *f*_AR1_. Compared with the antenna in Case 4, the antenna in Case 5 can further reduce the AR to less than 3 dB in the desired band. In addition, the current in TM_11_ mode (2.5 GHz) is also rotated on the patch surface due to the introduction of four C-shaped open slots and the approximately symmetric square structure, as verified in Figure 7. The current direction within a period of *t* = 0T, T/4, T/2, and 3T/4 changes clockwise in TM_11_ mode, indicating that the proposed antenna has LHCP characteristics. Due to the fact that another AR minimum of *f*_AR2_ is generated, the AR bandwidth of the proposed antenna is expanded.

### 2.3. Parametric Analysis 

To verify the operating mechanism and optimize the antenna performance, some parameters are further analyzed.

**Figure 8:**
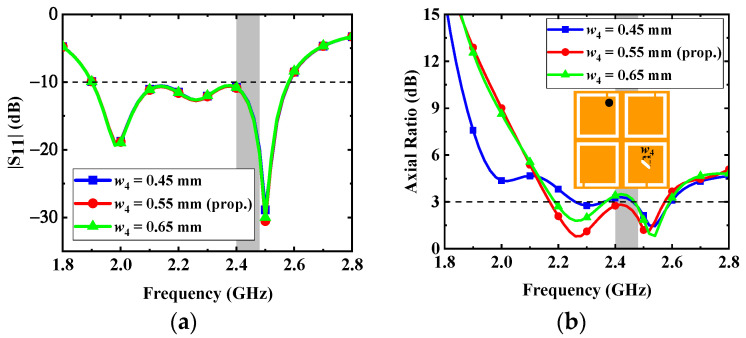
Effects of the dimension *w*_4_ on the antenna: (**a**) |S_11_| and (**b**) AR.

**Figure 9:**
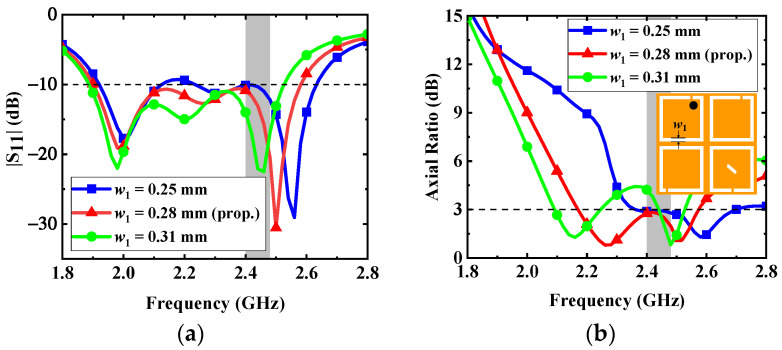
Effects of the dimension *w*_1_ on the antenna: (**a**) |S_11_| and (**b**) AR.

**Figure 10:**
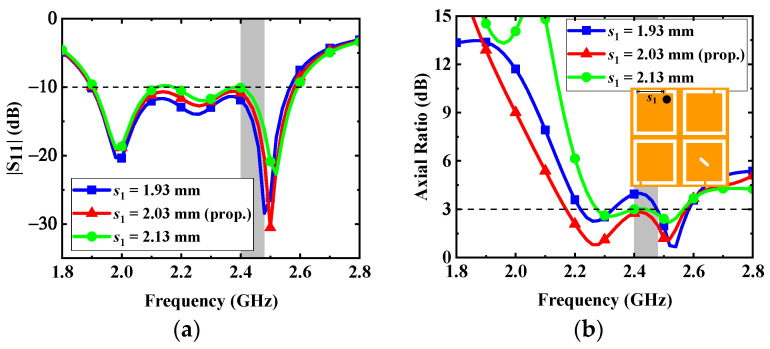
Effects of the dimension *s*_1_ on the antenna: (**a**) |S_11_| and (**b**) AR.

1. *Variations in the dimension (w_4_) of the diagonally truncated rectangular slot*: The effects of the dimension *w*_4_ of the rectangular slot truncated diagonally on the impedance matching and AR are shown in Figure 8. The CP purity of the proposed antenna is improved by the rectangular slot truncated diagonally, and the change of *w*_4_ is mainly related to the phase differences between the orthogonal degenerate modes TM_01_ and TM_10_, which have a great effect on *f*_AR1_. The *w*_4_ also has little effect on the S_11_ of the proposed antenna. According to Figure 8, to make the AR less than 3 dB in the desired band, the dimension *w*_4_ of the rectangular slot truncated diagonally is selected as 0.55 mm.

2. *Variations in the width (w_1_) of C-shaped slots*: The frequencies of three resonant modes are tuned by the width *w*_1_ of C-shaped slots in Figure 9. The introduction of four C-shaped slots enables the antenna to excite multiple resonant modes and achieve wide impedance bandwidth. With reference to Figure 9, the increase in the width *w*_1_ can extend the current path on the radiation patch; therefore, the resonant frequencies and AR can be shifted to the lower band. To make the bandwidth of the proposed antenna cover the desired band and maintain good AR performance, the width *w*_1_ is selected as 0.28 mm.

3. *Variations in the opening position s_1_ of the upper left C-shaped slots*: Since the opening position *s*_1_ of the upper left C-shaped slots is close to the coaxial feed, the variation of *s*_1_ can have an impact on the performance of the proposed antenna. The effects of the opening position *s*_1_ on the impedance matching and AR are shown in Figure 10. As seen in the figure, in order to make the AR bandwidth have good polarization purity, the opening position *s*_1_ is selected as 2.03 mm.

### 2.4. Safety Consideration

**Figure 11:**
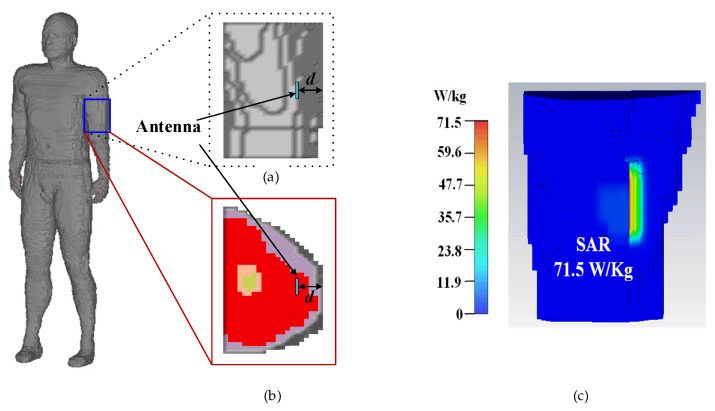
A total of 10 g of tissue is used for simulated SAR distribution with an input power of 1 W and an implant muscle depth of 4 mm at 2.45 GHz: (**a**) upper arm model, (**b**) cross section of the upper arm, and (**c**) SAR distribution.

The analysis of the SAR distribution is necessary to guarantee that the proposed antenna meet the requirements regulated by IEEE/ANSI. In theory [22], the implanted antenna has dual characteristics (i.e., if the antenna radiates electromagnetic (EM) waves strongly in one direction, it receives EM waves strongly in the same direction). For convenience, in the SAR calculation, the proposed antenna works as a transmitter instead of a wireless power receiver, radiating EM waves to the outside. When the input power of the proposed antenna is assumed to be 1 W, the maximum 10 g averaged SAR with an input power of 1 W at 2.45 GHz is illustrated in Figure 11, when the proposed antenna is implanted into the arm of the Hugo phantom. According to IEEE C95.1-2005 standard [23], the SAR for 10 g tissue should be less than 2 W/Kg. Through numerical computation, it can be inferred that the maximum input power satisfying the safety regulation should be less than 28 mW for radiation safety.

### 2.5. Antenna Measurement

**Figure 12:**
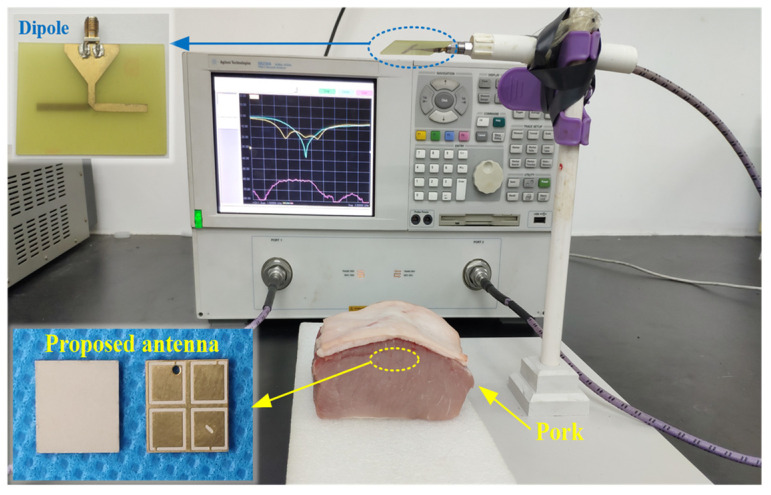
Photograph of the measurement setup with the fabricated implantable CP antenna.

**Figure 13:**
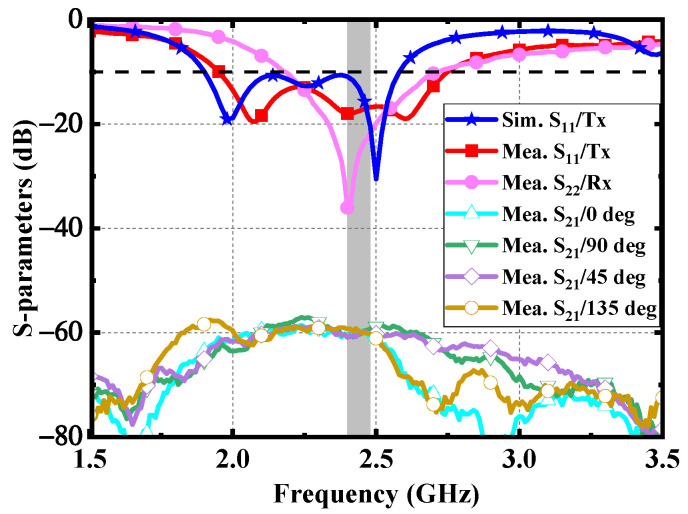
Measured results of the proposed antenna.

As shown in Figure 12, to validate the numerical performance of the proposed antenna, a prototype was fabricated and measured in fresh pork. Due to the fact that the electrical characteristics of pork and human tissues are very close, it is suitable for measuring the performance of implantable antennas. The proposed antenna was implanted in the muscle similar to the setup in the simulation. As shown in Figure 13, the measured impedance bandwidth is 33.2% (1.96–2.74 GHz). There is little difference between the results of simulation and measurement mainly due to the fabrication tolerance (e.g., the superstrate and the patch were not tightly bonded in the fabrication, and there could be some gaps between the implanted antenna and the pork) and the different electrical characteristics between the numerical phantom and fresh pork.

Furthermore, a linearly polarized dipole is employed as an external receiving antenna to evaluate the CP performance of the proposed antenna. As shown in Figure 13, the |S_21_| between the dipole and the proposed implantable antenna was measured at the different azimuth angles, such as 0°, 45°, 90°, and 135°. The distance between the dipole and the implanted antenna is 200 mm. The measured fluctuation degree of |S_21_| is within 3 dB in the ISM band of 2.4–2.48 GHz, which verifies that the proposed antenna has a high CP purity.

## 3. Rectenna Design

### 3.1. Rectifier Design

**Figure 14:**
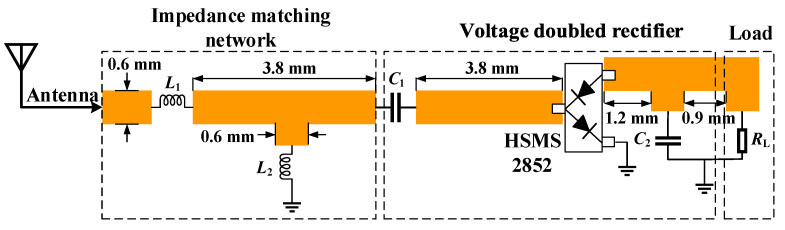
Schematic of the rectifier circuit: L_1_ = 2.7 nH, L_2_ = 1.8 nH, C_1_ = 100 pF, C_2_ = 100 pF, and R_L_ = 2 kΩ.

In the microwave wireless power transfer of IMDs, the RF power received via the implantable antenna needs to be converted into DC power through a rectifier circuit. To obtain a higher output DC voltage, the voltage doubled rectifier is adopted. As illustrated in Figure 14, the rectifier circuit consists of an impedance matching network, a voltage doubled rectifier, and a load.

**Figure 15:**
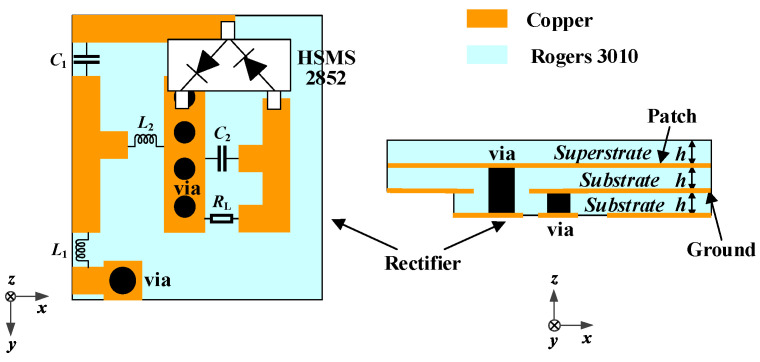
Configuration for integrating the rectifier circuit as a rectenna.

**Figure 16:**
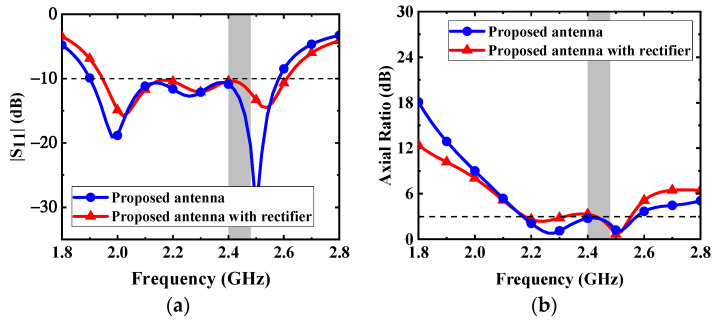
Effects of adding the rectifier layer on the proposed antenna: (**a**) |S_11_| and (**b**) AR.

Lumped components are welded in the circuit to maintain a compact size. Therefore, the rectifier circuit can be integrated under the proposed antenna. The antenna is directly connected to the rectifier circuit to form a rectenna. As shown in Figure 15, the received RF power from the proposed antenna is input into the rectifier circuit through a via, then a DC voltage is output to the load resistor. The source impedance of the proposed rectifier circuit is set as 50 Ω, integrating the proposed antenna and the circuit without impedance mismatch. The effect of adding the rectifier layer on the proposed antenna is illustrated in Figure 16. Owing to the fact that the equivalent permittivity around the proposed antenna decreases after adding the rectifier substrate layer, the resonant frequencies shift to a higher frequency band. Nevertheless, the proposed antenna can maintain good performance.

**Figure 17:**
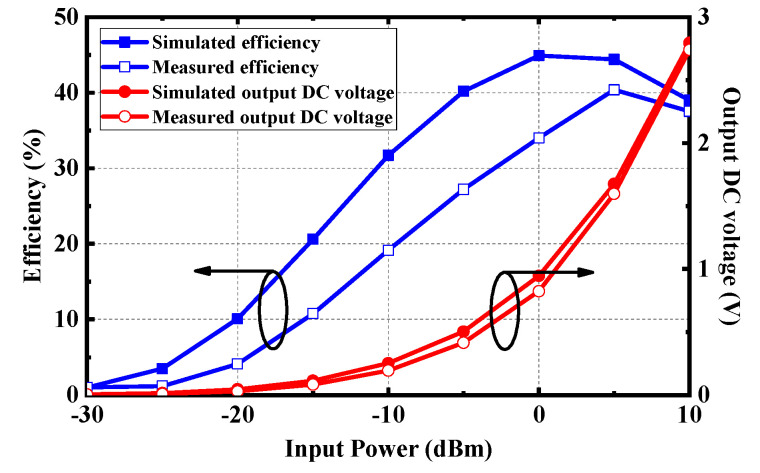
RF-to-DC conversion efficiency and output DC voltage of the rectifier.

The simulated output DC voltage and efficiency of the rectifier are demonstrated in Figure 17. With the low input power of –15 dBm, –10 dBm, and –5 dBm, the conversion efficiency can reach 21%, 32%, and 40%, respectively. In the measurement, the conversion efficiency can be obtained by measuring the output DC voltage with a voltmeter at different input power. As can be seen from Figure 16, the measured efficiency is a little lower than that of the simulation. The main reason is that the parasitic parameter effect of lumped elements at high frequency is more significant, and it is easy to deviate from the ideal frequency characteristic.

### 3.2. Wireless Power Transfer

**Figure 18:**
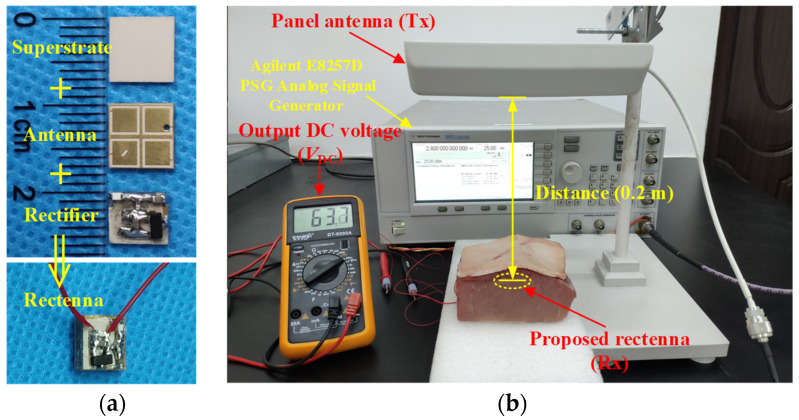
Photograph of measurement setup for wireless power transfer: (**a**) fabricated implantable rectenna and (**b**) measurement setup.

The prototype of the proposed rectenna, which acts as the receiving part of the microwave WPT system, is demonstrated in Figure 18a. In the RF energy harvesting system, the closer the distance between transmitting and receiving antennas, the higher the power transmission efficiency is. However, the electromagnetic radiation may affect health and safety as the antenna is in the proximity of the human body. Hence, the maximum permissible exposure (MPE) needs to be taken into consideration [24]. According to the Federal Communications Commission (FCC) standard, 10-W/m^2^ MPE should be followed at 2.4 GHz for uncontrolled exposure to an intentional radiator. The power flux density at a distance of *x* from the RF power source can be evaluated as

(1) $$S(x)=\frac{EIRP}{4\pi x^{2}}\leq 10\;{W/m}^{2}$$

It can be obtained that under the restriction of FCC standard for equivalent isotropically radiated power (EIRP), the minimum distance *x* of the transceiver antennas is 178 mm. Therefore, the distance of transmitting and receiving antennas in practical measurement is selected as 200 mm under safety considerations. The measurement setup of wireless power transfer is depicted in Figure 18b. An RF signal generator is employed to generate an analog signal with a frequency of 2.4 GHz and a power of 25 dBm, which is fed into a panel antenna with a gain of 9 dBi. The RF power can be received by the proposed rectenna implanted in the fresh pork from the panel antenna and then the DC power can be converted by the rectifier circuit. As shown in Figure 18b, the DC voltage output by the proposed implantable rectenna can be measured by the voltmeter as 63.7 mV. Note that since the transmitting antenna is a linearly polarized panel antenna, there is a 3-dB polarization mismatch loss between the transmitting antenna and the proposed rectenna. Here, the available linearly polarized panel antenna is employed for convenience, and a higher output DC voltage and efficiency can be obtained by using CP transmitting antennas in practical applications.

**Figure 19:**
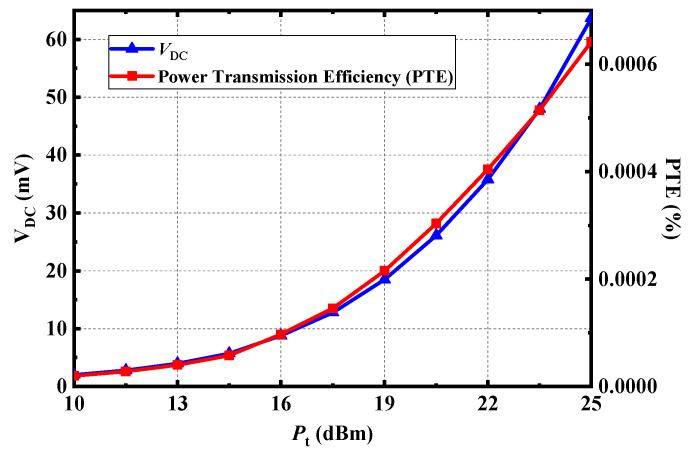
Measured output DC voltage and power transmission efficiency (PTE) of the proposed rectenna at 2.4 GHz.

Furthermore, the DC voltage output by the proposed rectenna and the power transmission efficiency of the system under different transmitting power can be tested in Figure 19. The power transmission efficiency of the whole system is low because of the high loss of receiving antenna implanted in biological tissues and the high loss of path. The higher the transmitting power, the higher the output DC voltage and transmission efficiency could be. Although the allowed EIRP under FCC rules is 36 dBm in the ISM-Bands, the transmitting power also needs to be limited. And the EIRP is ~34 dBm in the measurement, which satisfies the FCC standard.

## 4. Conclusions

A wideband circularly polarized implantable rectenna for microwave WPT has been presented. The comparison with previous CP implantable antennas is listed in Table 2. Although the antenna in [25] has high gain, its AR bandwidth is narrow, which makes the antenna sensitive to frequency deviation. Compared with the antennas in [20] and [25], the proposed CP antenna achieves wider impedance bandwidth and AR bandwidth while maintaining a compact size. Wide bandwidth allows antennas to collect RF power from multiple frequencies in the microwave WPT system. The performance of the proposed implantable rectenna and the power transfer system was tested under safety considerations. With wide bandwidth and compact size, the proposed implantable rectenna is suitable for microwave WPT in biomedical devices.

## Figures and Tables

**Table 1:** Optimized antenna dimensions (UNIT: mm).

Parameter	Value	Parameter	Value	Parameter	Value
*L*	7.5	*w*_1_	0.28	*w*_2_	0.15
*w*_3_	0.7	*w*_4_	0.55	*l*_1_	2.87
*l*_2_	2.74	*s*_1_	2.03	*s*_2_	0.56
*s*_3_	0.2	*s*_4_	1.22	*h*	0.635

**Table 2:** Performance comparison with previous arts.

Ref.	Frequency (MHz)	Volume (mm^3^)	Bandwidth		Gain (dBi)	Implant Depth
			(|S_11_| < −10 dB)	(AR < 3 dB)		
[25]	2450	1016	37.5%	5.3%	−15.9	4 mm
[25]	2400	121.97	21.5%	15.8%	−33	3 mm
[20]	915	153.67	3.9%	1.2%	−29.2	4 mm
This Work	2400	71.4	30.4%	16.9%	−32.8	12 mm

## Data Availability

The data presented in this study are available on request from the corresponding author. The data are not publicly available due to potential patent application.
